# Development and construction of a cataract risk prediction model based on biochemical indices: the National Health and Nutrition Examination Survey, 2005–2008

**DOI:** 10.3389/fmed.2024.1452756

**Published:** 2024-10-21

**Authors:** Guoqing Wang, Xiang-Long Yi

**Affiliations:** ^1^Department of Ophthalmology, First Affiliated Hospital of Xinjiang Medical University, Urumqi, Xinjiang, China; ^2^Xinjiang Medical University, Urumqi, Xinjiang, China

**Keywords:** cataract, blood biochemical indicators, prediction model, nomogram, machine learning

## Abstract

**Purpose:**

The aim of this study is to develop and validate a novel multivariable prediction model capable of accurately estimating the probability of cataract development, utilizing parameters such as blood biochemical markers and age.

**Design:**

This population-based cross-sectional study comprised 9,566 participants drawn from the National Health and Nutrition Examination Survey (NHANES) across the 2005–2008 cycles.

**Methods:**

Demographic information and laboratory test results from the patients were collected and analyzed using LASSO regression and multivariate logistic regression to accurately capture the influence of biochemical indicators on the outcomes. The SHAP (Shapley Additive Explanations) scale was employed to assess the importance of each clinical feature, excluding age. A multivariate logistic regression model was then developed and visualized as a nomogram. To assess the model’s performance, its discrimination, calibration, and clinical utility were evaluated using receiver operating characteristic (ROC) curves, 10-fold cross-validation, Hosmer-Lemeshow calibration curves, and decision curve analysis (DCA), respectively.

**Results:**

Logistic regression analysis identified age, erythrocyte folate (nmol/L), blood glucose (mmol/L), and blood urea nitrogen (mmol/L) as independent risk factors for cataract, and these variables were incorporated into a multivariate logistic regression-based nomogram for cataract risk prediction. The area under the receiver operating characteristic (ROC) curve (AUC) for cataract risk prediction was 0.917 (95% CI: 0.9067–0.9273) in the training cohort, and 0.9148 (95% CI: 0.8979–0.9316) in the validation cohort. The Hosmer-Lemeshow calibration curve demonstrated a good fit, indicating strong model calibration. Ten-fold cross-validation confirmed the logistic regression model’s robust predictive performance and stability during internal validation. Decision curve analysis (DCA) demonstrated that the nomogram prediction model provided greater clinical benefit for predicting cataract risk when the patient’s threshold probability ranged from 0.10 to 0.90.

**Conclusion:**

This study identified blood urea nitrogen (mmol/L), serum glucose (mmol/L), and erythrocyte folate (mmol/L) as significant risk factors for cataract. A risk prediction model was developed, demonstrating strong predictive accuracy and clinical utility, offering clinicians a reliable tool for early and effective diagnosis. Cataract development may be delayed by reducing levels of blood urea nitrogen, serum glucose, and erythrocyte folate through lifestyle improvements and dietary modifications.

## 1 Introduction

Cataracts, characterized by the clouding of the lens, are a leading cause of vision impairment and blindness among older adults worldwide (1). In China, the prevalence of cataracts among individuals aged 45–89 exceeds 22% (2). Further research indicates that the cataract prevalence among individuals aged 60 and older ranges between 53 and 58% (3). With population growth and aging, the incidence of cataracts and the demand for cataract surgeries are expected to rise steadily (4). Although cataract surgery significantly improves vision, it remains prohibitively expensive, and many low-income countries face a shortage of skilled surgeons (5). Reducing cataract incidence is essential. Cataracts are associated with multiple factors, including smoking, diabetes, UV exposure, and blood metabolites (6). Identifying and targeting modifiable risk factors can substantially reduce the health and economic burden of cataracts.

Aging is the predominant factor influencing cataract development; however, additional factors also contribute to their onset (7). Numerous researchers have explored the impact of nutritional status on cataract formation and the potential use of biochemical markers to assess the risk of cataractogenesis, as these parameters can be modified by lifestyle changes (8, 9). Blood biochemical markers serve as key indicators of the body’s overall metabolic state (10). Therefore, we aim to develop a logistic regression model incorporating blood biochemical markers and age to visualize the components contributing to cataract risk via a nomogram. However, to the best of our knowledge, no existing model currently predicts cataracts based on blood biochemical markers and age.

This article presents the findings of a cross-sectional study using data from the National Health and Nutrition Examination Survey (NHANES) conducted between 2005 and 2008. The aim of our study was to develop and validate a novel multivariate predictive model to accurately assess the probability of cataract onset based on blood biochemical markers and age. Additionally, we sought to explore the potential causes of cataracts.

## 2 Materials and methods

### 2.1 Data source and study population

**Figure 1:**
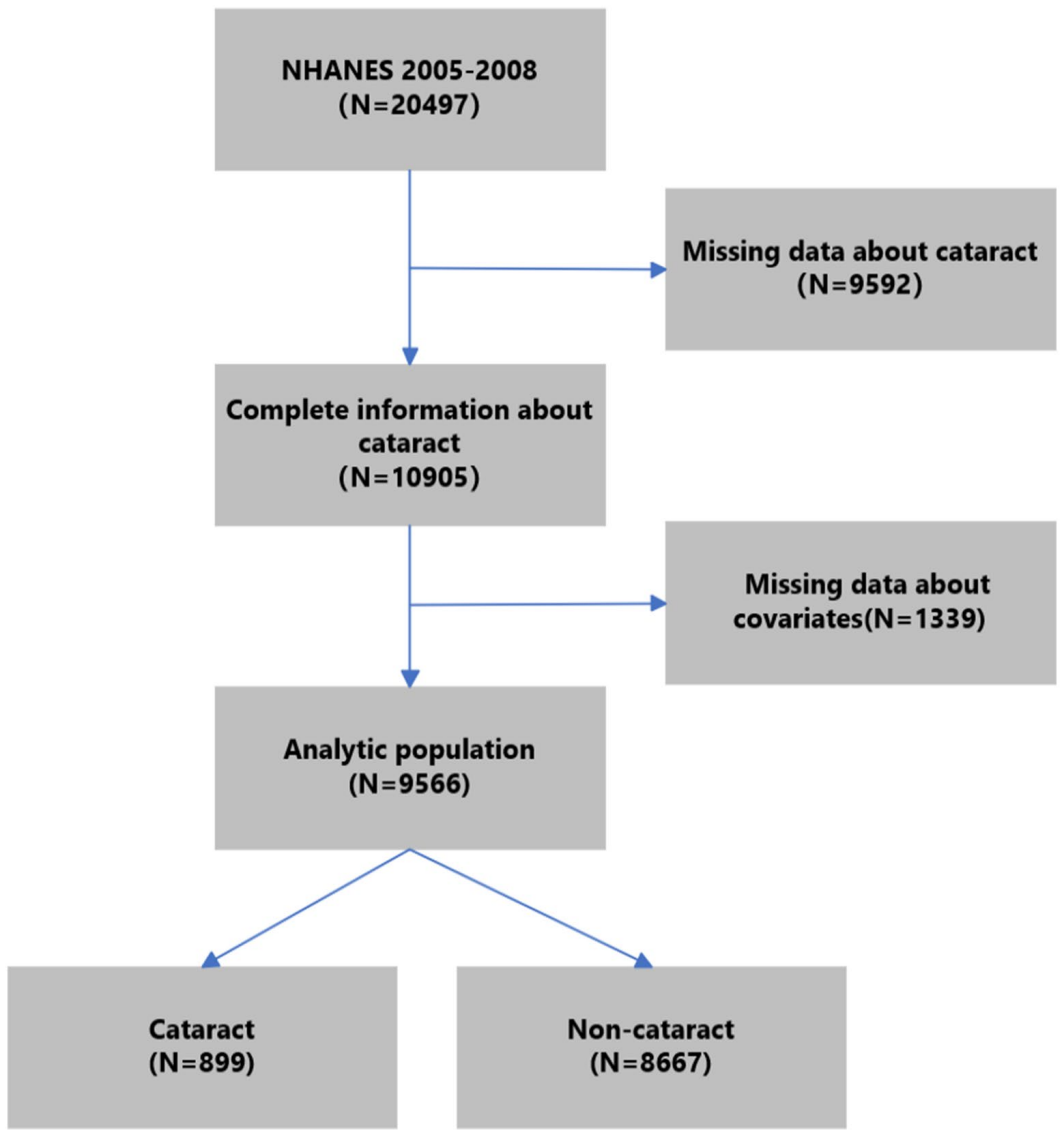
Flow chart of the study population.

NHANES is an extensive nationwide survey conducted by the National Center for Health Statistics. Its purpose is to evaluate the health and nutritional condition of the American people. It is a department of the U.S. Centers for Disease Control and Prevention. The survey data in NHANES were organized in a biennial style. We utilized data from two consecutive survey cycles (2005–2006 and 2007–2008) about cataracts. Of all 20,497 participants in NHANES 2005–2008, we excluded those without complete information on cataracts (*n* = 9,592). Further, we excluded participants under 20 years old without complete information on other covariates (*n* = 1,339). Finally, 9,566 subjects were included in the analytic population. The process of participant selection is summarized in Figure 1.

### 2.2 Cataract assessment

Consistent with other epidemiological research, a cataract operation was used as a surrogate for a cataract (11). The occurrence of a cataract operation was ascertained by inquiring participants about their history of undergoing a cataract operation. (VIQ071), with responses limited to “yes” or “no.” If the response was affirmative, the subject was diagnosed with a cataract (12).

### 2.3 Covariates assessment

According to previous epidemiological studies concerning cataracts (13), potential confounding factors studied in the current work included sociodemographic factors (gender, age, race) and blood biochemical parameters. The sociodemographic characteristics were obtained using self-reported questionnaires, which included information on gender (male or female), age (continuous), and race (non-Hispanic white, non-Hispanic black, Mexican American, etc.). The source of the blood biochemical parameter specimen is serum. The serum specimens undergo processing, storage, and shipment to the Collaborative Laboratory Services for analysis. The NHANES Laboratory/Medical Technologists Procedures Manual (LPM) provides in-depth instructions on how to collect and prepare specimens. The NHANES QA/QC processes adhere to the requirements set by the 1988 Clinical Laboratory Improvement Act. The NHANES Laboratory/Medical Technologists Procedures Manual (LPM) provides comprehensive guidance for quality assurance and quality control (QA/QC) procedures. Refer to the General Documentation of the Laboratory Data file for comprehensive quality assurance and quality control techniques.

The subsequent blood biochemical values were gathered from patients with cataracts for further study. The laboratory examined the following values: albumin (g/L), alanine aminotransferase (ALT) (U/L), aspartate aminotransferase (AST) (U/L), alkaline phosphatase (U/L), and blood urea nitrogen (mmol/L). Blood calcium concentrations were measured in millimoles per liter (mmol/L), cholesterol levels in millimoles per liter (mmol/L), and bicarbonate, creatinine, and gamma glutamyltransferase concentrations in millimoles per liter (mmol/L), micromoles per liter (μmol/L), and units per liter (U/L), respectively. The serum’s glucose concentration was measured in millimoles per liter (mmol/L), while the iron content was measured in micromoles per liter (umol/L) and needed to be kept in a refrigerator. The concentration of bilirubin in the blood is measured in micromoles per liter (umol/L). The following measurements are provided in the given units: total protein concentration (g/L), triglycerides (mmol/L), uric acid (mmol/L), sodium (mmol/L), potassium (mmol/L), chloride (mmol/L), osmolality (mmol/kg), globulins (g/L), C-reactive proteins (mg/dL), erythrocyte folate (mmol/L), serum folate (mmol/L), and glycated hemoglobin (%).

## 3 Statistical analysis

The median (interquartile range) was employed to represent continuous data, while categorical data were expressed as number (percentage) (14, 15). Comparisons between cataract and non-cataract groups were conducted using statistical tests such as the unpaired t-test, Wilcoxon rank-sum test, Pearson Chi-square test, or Fisher’s exact test, as appropriate. Cases from the NHANES dataset were randomly allocated into a training set (*n* = 6,696) and a validation set (*n* = 2,870) in a 7:3 ratio. The outcome variable for this study was cataract status. To manage data dimensionality and predictor selection, the researchers employed the least absolute shrinkage and selection operator (LASSO) regression and multivariable logistic regression (16). Multivariable logistic regression analysis was used to develop a predictive model and a nomogram of cataract (17). The model’s discriminative ability was assessed by calculating the area under the curve (AUC) (18). To enhance the estimation of model performance, 10-fold cross-validation was employed for evaluation. The model’s calibration was assessed using the Hosmer-Lemeshow test and calibration curve, while its clinical utility was evaluated through decision curve analysis (DCA) (19). All statistical analyses were performed using R software (version 4.3.2; R Foundation for Statistical Computing, Vienna, Austria) and Python (version 3.12). A significance threshold of *p* < 0.05 was applied to determine statistical significance.

LASSO regression (Least Absolute Shrinkage and Selection Operator) efficiently integrates variable selection with regularization, enhancing both the predictive accuracy and interpretability of statistical models. Through the introduction of an L1 penalty, LASSO reduces specific coefficients to zero, thus enabling efficient variable selection. The optimal lambda (*λ*) is typically determined via 10-fold cross-validation, aiming to minimize prediction error while balancing model complexity and fit. In this study, 20-fold cross-validation was employed, which, despite the higher computational costs, produces more stable and accurate model evaluations.

The analysis commenced with data preprocessing, wherein categorical variables such as sex, diagnosis, and race were transformed into factor variables to ensure appropriate handling during modeling. Numerical variables were subsequently normalized using a min-max scaling function, which transformed each variable into a range of [0,1]. This normalization is critical in LASSO regression, as the model is sensitive to the scale of the input variables. The transformation was applied to all numeric variables within the dataset using the mutate_if function within a pipeline, and the resultant dataset was converted into a data frame for further processing. To ensure reproducibility, a random seed was set [set.seed (123)], and the preprocessed data was partitioned into a matrix of predictors (*x*) and a response vector (*y*). The response vector y was further converted to numeric form to be compatible with the modeling functions. The LASSO regression was executed using the glmnet function, specifying a binomial family to accommodate the binary nature of the outcome variable. The function was configured to evaluate 1,000 distinct values of the regularization parameter lambda (n lambda = 1,000), enabling the model to thoroughly explore the regularization path. This extensive range of lambda values ensures that the model can identify the optimal level of penalization, balancing model complexity with predictive performance. Following the initial fitting of the LASSO model, the regularization path was visualized using a plot of the model coefficients against the logarithm of lambda. This plot facilitates understanding of how the coefficients shrink as the penalization increases, and which variables remain significant across varying levels of lambda. To validate the model and prevent overfitting, a 20-fold cross-validation was conducted using the cv. glmnet function. This process involves partitioning the data into 20 subsets, fitting the model on 19 subsets, and validating it on the remaining one. This procedure is repeated 20 times, ensuring that each subset serves as a validation set once. The cross-validation results were plotted to visualize the relationship between lambda and the cross-validated error, aiding in the selection of the most appropriate lambda value. Two key lambda values were identified from the cross-validation results: Lambda. min: The lambda value that minimizes the cross-validated mean squared error (MSE), representing the point at which the model achieves the best predictive accuracy. Lambda.1se: The lambda value that is one standard error above the minimum MSE. This value typically results in a more parsimonious model, as it provides a simpler model with fewer predictors, while still maintaining a reasonable level of accuracy. Finally, the model coefficients corresponding to lambda. 1se were extracted using the coef function. These coefficients indicate which variables are most influential in predicting the outcome, offering insights into the underlying relationships within the data.

For multivariable logistic regression:variable selection criteria are based on significance testing (*p* < 0.05). LASSO-screened variables were included in the multivariable logistic regression, and variables with *p* less than 0.05 were selected for the prediction model.

## 4 Results

### 4.1 Patient characteristics

**Figure 2:**
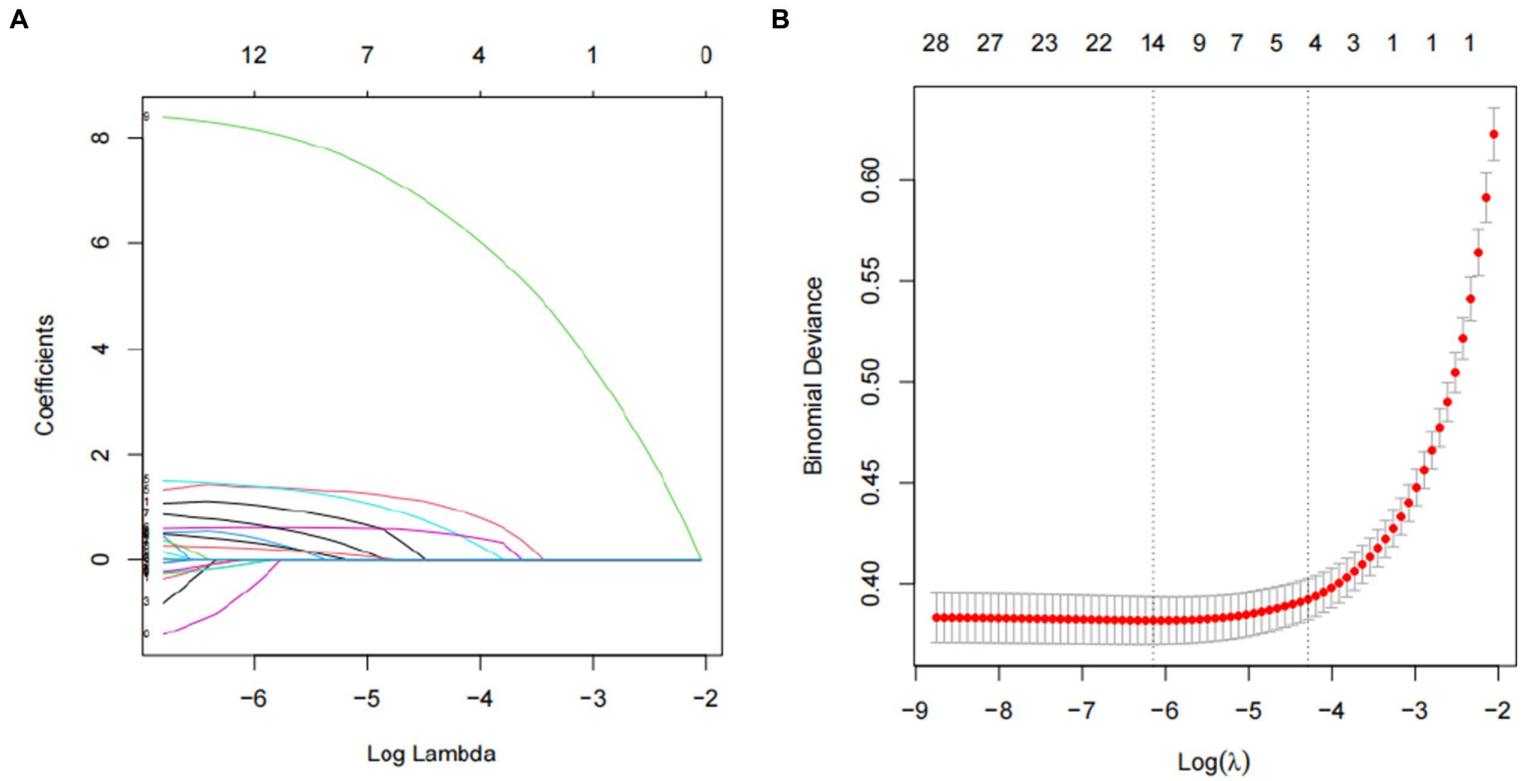
Predictor selection using the LASSO regression analysis with twenty fold cross-validation. (A) Tuning parameter (lambda) selection of deviance in the LASSO regression based on the minimum criteria (left dotted line) and the 1-SE criteria (right dotted line). (B) A coefficient profile plot was created against the log (lambda) sequence. In the present study, predictor’s selection was according to the 1-SE criteria (right dotted line), where 5 nonzero coefficients were selected. LASSO, least absolute shrinkage and selection operator; SE, standard error.

Out of the individuals involved in the study, 9.4% (899 out of 9,556) were diagnosed with cataracts. Table 1 displays the demographic and clinical characteristics of the individuals who participated in the study. Out of the 30 variables obtained from patients, 5 were chosen based on non-zero coefficients produced by LASSO regression analysis (Figure 2).

**Table 1:** Demographic and clinical characteristics of study participants.

Characteristic	Total (*n* = 9,566)	Cataract (*n* = 899)	Non-cataract (*n* = 8,667)	*p*
Gender, *n* (%)				0.089
Male	4,637 (48)	411 (46)	4,226 (49)	
Female	4,929 (52)	488 (54)	4,441 (51)	
Age, Median (Q1,Q3)	48 (34, 64)	78 (70, 80)	46 (33, 61)	< 0.001
Ethnicity, *n* (%)				< 0.001
Mexican American	1821 (19)	72 (8)	1749 (20)	
Other Hispanic	719 (8)	56 (6)	663 (8)	
Non-Hispanic White	4,678 (49)	633 (70)	4,045 (47)	
Non-Hispanic Black	1957 (20)	112 (12)	1845 (21)	
Other Race - Including Multi-Racial	391 (4)	26 (3)	365 (4)	
Albumin (g/L), Median (Q1,Q3)	42 (40, 44)	41 (39, 44)	42 (40, 44)	< 0.001
alanine aminotransferase (ALT) (U/L), Median (Q1,Q3)	21 (16, 28)	19 (15, 24)	21 (17, 29)	< 0.001
Asparate aminotransferase (AST) (U/L), Median (Q1,Q3)	23 (20, 28)	24 (21, 28)	23 (20, 28)	0.001
Alkaline phosphotase (U/L), Median (Q1,Q3)	67 (55, 82)	71 (59, 86)	67 (55, 82)	< 0.001
Blood urea nitrogen (mmol/L), Median (Q1,Q3)	4.28 (3.21, 5.36)	6.07 (4.28, 7.85)	4.28 (3.21, 5.36)	< 0.001
Total calcium (mmol/L), Median (Q1,Q3)	2.35 (2.3, 2.42)	2.35 (2.3, 2.42)	2.35 (2.3, 2.42)	0.319
Cholesterol (mmol/L), Median (Q1,Q3)	5.04 (4.37, 5.82)	4.89 (4.19, 5.77)	5.07 (4.4, 5.82)	< 0.001
Bicarbonate (mmol/L), Median (Q1,Q3)	25 (23, 26)	25 (24, 27)	25 (23, 26)	< 0.001
Creatinine (μmol/L), Median (Q1,Q3)	76.91 (63.65, 89.28)	88.4 (72.49, 106.08)	74.26 (63.65, 88.4)	< 0.001
Gamma glutamyl transferase (U/L), Median (Q1,Q3)	21 (15, 32)	20 (15, 30)	21 (15, 32)	0.18
Glucose, serum (mmol/L), Median (Q1,Q3)	5.16 (4.72, 5.77)	5.61 (5, 6.61)	5.11 (4.66, 5.72)	< 0.001
Iron, refigerated (umol/L), Median (Q1,Q3)	14.3 (10.7, 18.75)	13.6 (10.6, 18.1)	14.5 (10.7, 18.8)	0.003
Lactate dehydrogenase LDH (U/L), Median (Q1,Q3)	128 (113, 146)	140 (122, 160)	127 (112, 144)	< 0.001
Phosphorus (mmol/L), Median (Q1,Q3)	1.23 (1.1, 1.32)	1.23 (1.1, 1.32)	1.23 (1.1, 1.32)	0.595
Bilirubin, total (umol/L)(umol/L), Median (Q1,Q3)	11.97 (8.55, 15.39)	11.97 (10.26, 15.39)	11.97 (8.55, 15.39)	< 0.001
Total protein (g/L), Median (Q1,Q3)	71 (68, 74)	70 (68, 74)	71 (68, 75)	< 0.001
Triglycerides (mmol/L), Median (Q1,Q3)	1.42 (0.94, 2.2)	1.55 (1.05, 2.23)	1.41 (0.93, 2.2)	< 0.001
Uric acid (umol/L), Median (Q1,Q3)	315.2 (261.7, 374.7)	333.1 (279.6, 395.55)	315.2 (261.7, 374.7)	< 0.001
Sodium (mmol/L), Median (Q1,Q3)	139 (138, 141)	140 (138, 141)	139 (138, 140)	< 0.001
Potassium (mmol/L), Median (Q1,Q3)	3.95 (3.7, 4.2)	4.1 (3.8, 4.3)	3.9 (3.7, 4.1)	< 0.001
Chloride (mmol/L), Median (Q1,Q3)	104 (102, 106)	104 (101, 105)	104 (102, 106)	< 0.001
Osmolality (mmol/Kg), Median (Q1,Q3)	278 (275, 281)	281 (277, 284)	278 (275, 281)	< 0.001
Globulin (g/L), Median (Q1,Q3)	29 (26, 32)	29 (26, 32)	29 (27, 32)	0.125
C-reactive protein(mg/dL), Median (Q1,Q3)	0.21 (0.08, 0.49)	0.25 (0.1, 0.56)	0.21 (0.08, 0.49)	< 0.001
RBC folate (nmol/L), Median (Q1,Q3)	840.3 (586.6, 1,220)	1112.1 (758.8, 1,620)	820 (576, 1184.6)	< 0.001
Serum folate (nmol/L), Median (Q1,Q3)	31.5 (21.5, 45.9)	43.9 (29.5, 71.05)	30.6 (21, 44)	< 0.001
Glycohemoglobin (%), Median (Q1,Q3)	5.4 (5.2, 5.8)	5.7 (5.4, 6.2)	5.4 (5.1, 5.7)	< 0.001

### 4.2 Identification of the risk factors for cataract

The variables consisted of blood urea nitrogen (mmol/L), blood glucose (mmol/L), erythrocyte folate (mmol/L), serum folate (mmol/L), and age. The logistic regression prediction model was created using a multivariable method, incorporating the five factors chosen by LASSO regression as independent variables. The research’s findings demonstrate that blood urea nitrogen (mmol/L), glucose (mmol/L), serum (mmol/L), erythrocyte folate (nmol/L), and age have been identified as risk factors for cataract. These results are presented in Table 2.

**Table 2:** Multivariate logistic regression was used to analyze the influencing factors of cataract.

variable	*β*	SE	*z* value	OR	2.5–97.5%CI	*p* value
Blood urea nitrogen (mmol/L)	0.041	0.016	2.563	1.042	1.009–1.074	<0.001
Glucose, serum (mmol/L)	0.069	0.017	4.168	1.072	1.036–1.107	0.01
RBC folate (nmol/L)	0.0003	0	3.281	1	1.0001–1.0004	<0.001
Serum folate (nmol/L)	0.0016	0.001	1.054	1.002	0.998–1.004	0.291
Age	0.134	0.005	28.811	1.143	1.133–1.152	<0.001

### 4.3 Comparison of predictive influence

It is crucial to compare the impact of biochemical indicators with the influence of age, given that age remains the most significant predictor of cataracts. When age was used as the sole predictor in this study’s dataset, the area under the ROC curve (AUC) was 0.9167 (95% CI: 0.9066–0.9267) in the training set and 0.904 (95% CI: 0.8853–0.9228) in the validation set. While age alone demonstrated robust predictive performance, incorporating models identified through LASSO and multivariate logistic regression further enhanced predictive accuracy. In this study, the AUC for the training set was 0.917 (95% CI: 0.9067–0.9273), and for the validation set, the AUC was 0.9148 (95% CI: 0.8979–0.9316).

### 4.4 Utilizing SHAP to highlight variable importance

To facilitate the visual interpretation of the selected variables, we employed SHAP (20) to elucidate the specific contributions of these variables to the model’s prediction of cataract formation. Figure 8 highlights the 19 most significant features in the logistic regression model, which was developed using 29 variables. Each feature’s contribution to the outcome is represented by colored dots along the significance line, with red indicating high-risk values and blue representing low-risk values. Among the top five features, elevated levels of blood urea nitrogen, serum folate, erythrocyte folate, osmolality, and potassium were associated with an increased risk of age-related cataract formation. Figure 9 presents the ranking of the 19 risk factors, evaluated by the mean absolute SHAP value, with the SHAP value on the *X*-axis reflecting each factor’s importance in the predictive model. Without variable screening, the ROC curve for the test set was 0.8 when all variables were included in the model, and 0.73 when only blood urea nitrogen was included. After applying the stacked formula sequentially, model performance did not improve with the inclusion of the third variable, erythrocyte folate. The area under the ROC curve for the test set was 0.77, decreasing slightly to 0.76 following the inclusion of erythrocyte folate. Two variables, blood urea nitrogen and erythrocyte folate, were consistently selected through LASSO and multivariate logistic regression screening, indicating their significant impact on cataract prognosis. However, based on the SHAP scores, blood urea nitrogen, serum folate, and erythrocyte folate were ranked 1st, 2nd, and 3rd, respectively, while serum glucose was ranked 11th in terms of importance. In summary, the model constructed using variables identified through LASSO and multivariate logistic regression screening proved to be feasible.

**Figure 3:**
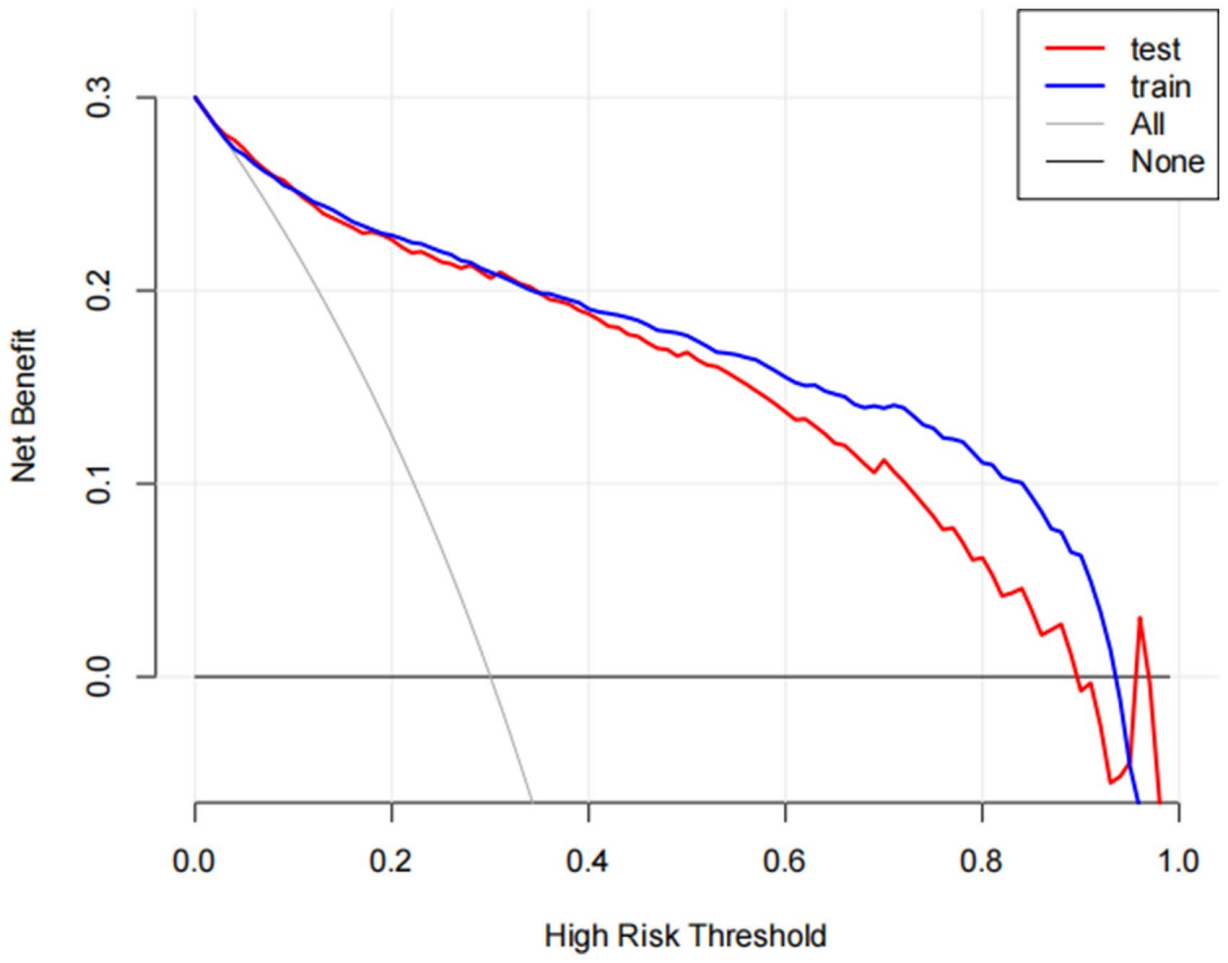
The benefit curve represented by the prediction model. The *y*-axis indicates the overall net benefit, which is calculated by summing the benefits (true positive results) and subtracting the harms (false positive results). The *x*-axis indicates the threshold that used to decide whether it is high risk to have cataracts. All: net benefit curve when all cataract patients are treated. None: net benefit curve when all cataract patients are not treated.

### 4.5 Construction of predictive model for cataract

**Figure 4:**
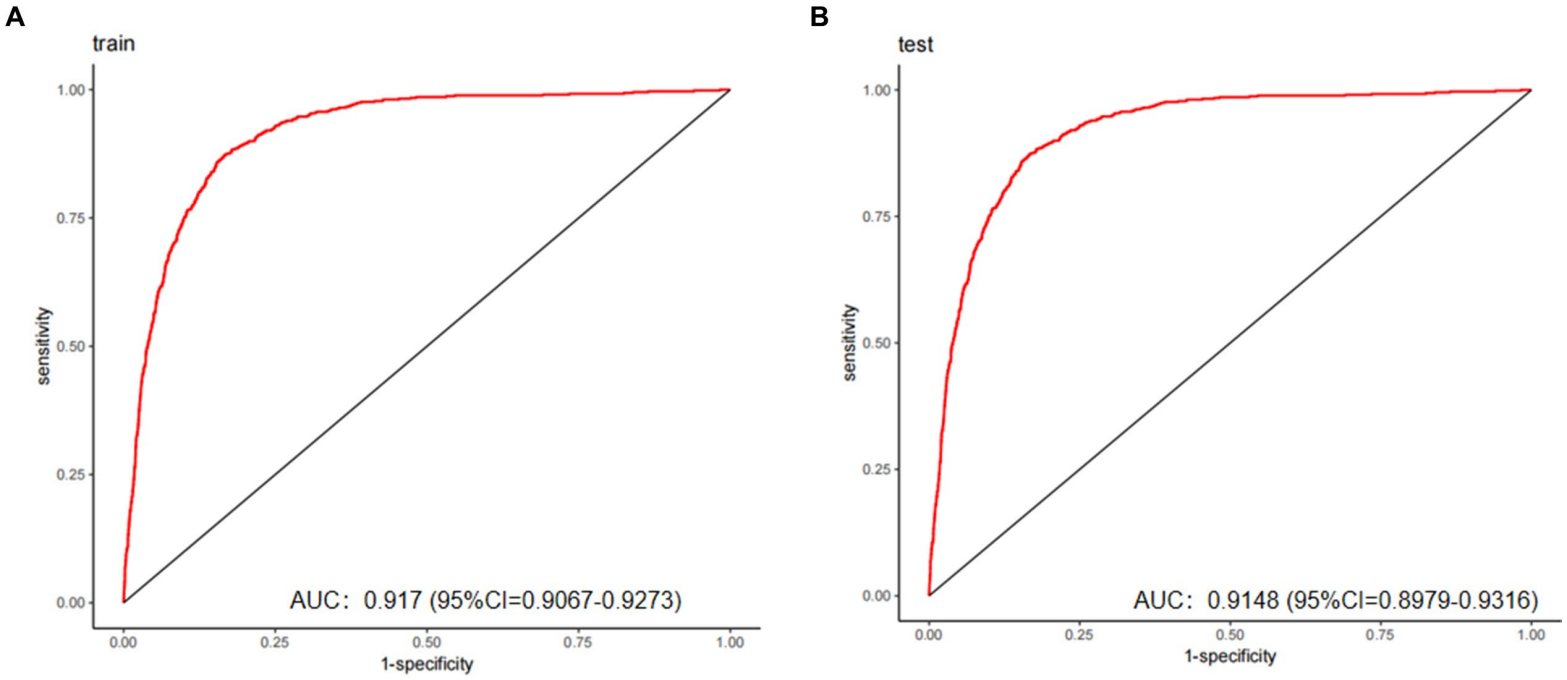
The predictive model’s performance was assessed using ROC curves for both the training (A) and validation (B) groups, yielding AUC values of 0.917 and 0.9148, respectively. These results demonstrate good discriminative capacity and excellent generalizability.

**Figure 5:**
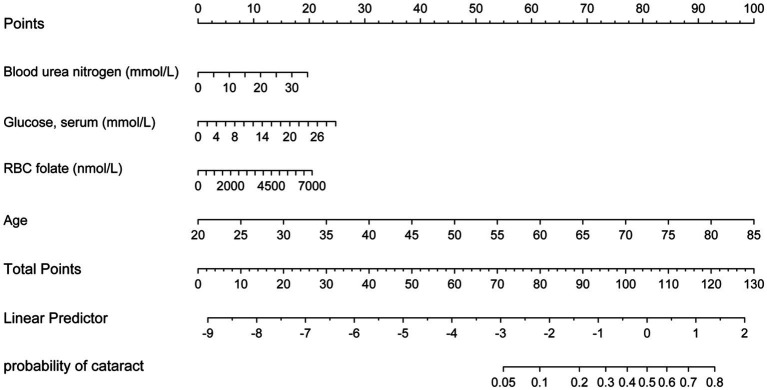
Nomogram for predicting cataract risk and its algorithm. First, a point was found for each variable of a people who may have cataracts on the uppermost rule; then all scores were added together and the total number of points were collected. Finally, the corresponding predicted probability of people who may have cataracts was found on the lowest rule.

**Figure 6:**
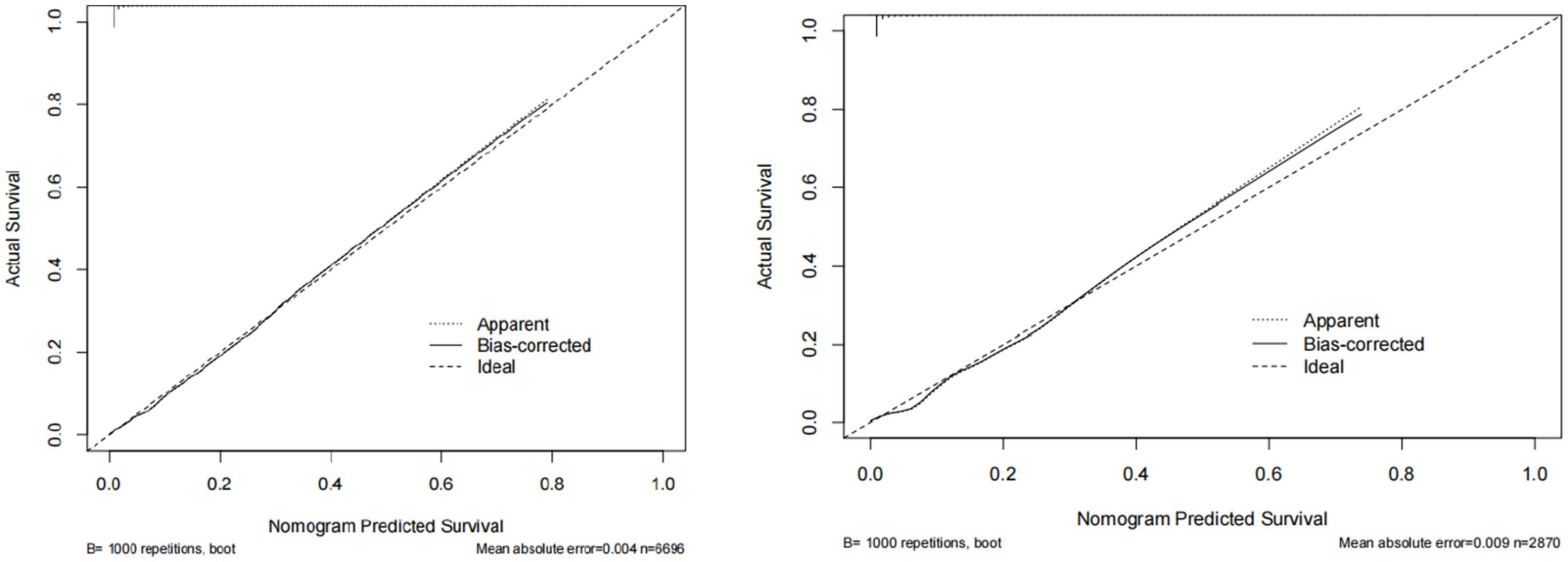
The calibration curve of predictive nomograms for predicting cataracts. The nomogram shows the predicted probability on the *x*-axis and the actual probability on the *y*-axis.

**Figure 7:**
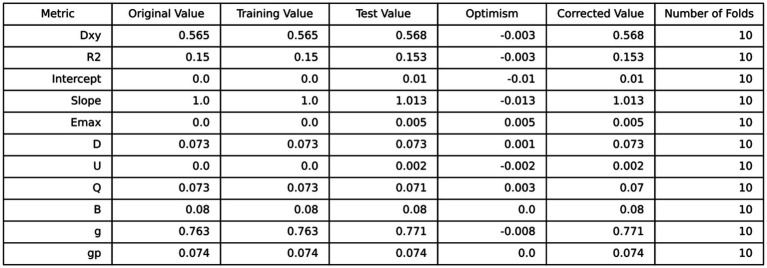
Provides a summary of the results from 10-fold cross-validation.

Based on the four variables indicated above that were chosen using the LASSO regression approach and the logistic regression technique, multivariable logistic regression analysis was carried out to create a predictive model for cataract. The differentiation of the cataract risk prediction model was assessed using the ROC curve. The training group’s AUC was 0.917 (95%CI = 0.9067–0.9273) and the validation group’s was 0.9148 (95%CI = 0.8979–0.9316), according to the data (Figure 4). A nomogram was created in order to depict the predictive model, offering a useful customized tool for assessing the probability of cataract development (Figure 5). The suggested model (Figure 6) has good calibration. For the Hosmer-Lemeshow test, a *p*-value of less than 0.05 is typically seen as indicating a poor model fit and a significant discrepancy between the predicted and true values. However, this study’s huge sample size is associated with the HL test results (21). With bigger and larger sample sizes, it is more likely that simply uncorrelated disparities between estimated and true probability will result in the rejection of the perfect fit hypothesis since the power of classic goodness-of-fit tests grows with sample size (22). As a result, an HL test *p*-value of less than 0.05 does not always signify a poor model fit. In this study, the relatively small deviation of the calibration curves from the reference line indicates that the fit between predicted and observed values is not statistically significantly biased and is therefore highly credible. To further assess model calibration, we computed the Brier score (23), a metric that evaluates the accuracy of probabilistic predictions, particularly for binary outcomes. A Brier score of 0.057 in the training set indicates strong model calibration, reflecting the model’s accurate probabilistic performance. We utilized 10-fold cross-validation for model evaluation, and the resulting performance metrics are presented in Figure 7. Based on these results, our 10-fold cross-validation analysis confirms that the logistic regression model exhibits moderate-to-strong predictive ability and is likely to perform robustly in external validation studies.

DCA was also carried out to evaluate its clinical utility (Figure 3). In decision curve analysis (DCA), the model optimizes true positive rates while minimizing false positives, confirming its capacity to improve clinical decision-making by delivering considerable net benefit across a range of threshold probabilities. The decision curve consistently remains above the “None” line (representing no intervention) across a broad spectrum of threshold probabilities, demonstrating a positive net benefit. This illustrates the model’s clinical utility in identifying high-risk patients likely to benefit from intervention. Conversely, red and blue curves falling below the “None” line at higher threshold probabilities suggest that treating all patients results in unnecessary interventions, thereby diminishing net benefit.

**Figure 8:**
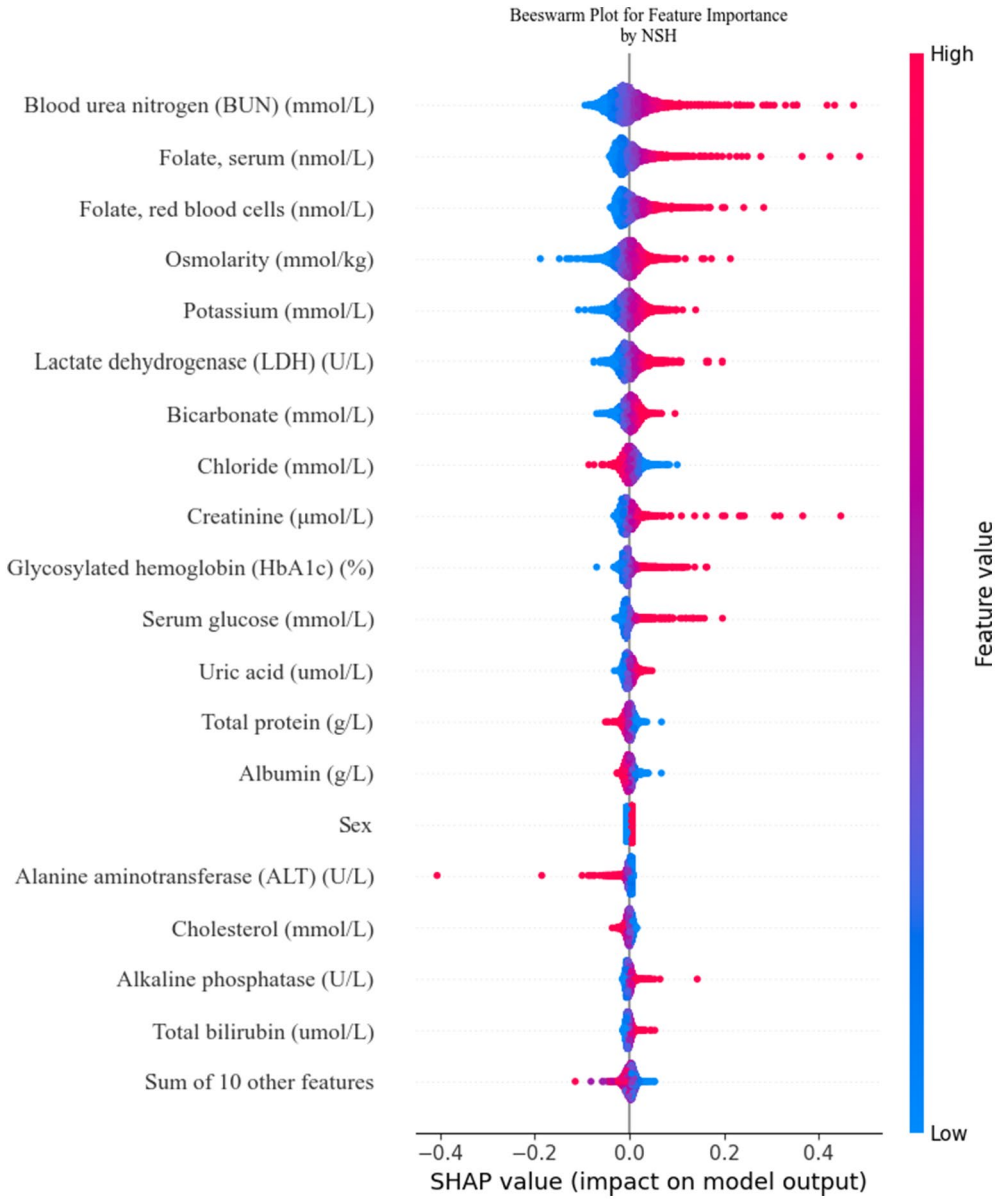
Feature contributions in SHAP: each line represents a feature, with the SHAP value plotted on the *x*-axis. Red dots indicate higher feature values, while blue dots indicate lower feature values. The spread of the dots along the x-axis illustrates the impact of each feature on the model’s prediction.

Decision curve analysis demonstrates that the nomogram provides optimal predictive performance for cataract risk within high-risk thresholds of 0.10 to 0.90, delivering superior net benefit compared to treating all patients or none. At a threshold of 0.4, where patients with a 40% predicted probability are classified as high-risk and receive treatment, the model yields a net benefit of 0.2. This signifies that 20 out of every 100 patients benefit from treatment without undergoing unnecessary interventions. At a threshold of 0.5, the net benefit decreases to 0.15, indicating that 15 out of every 100 patients benefit from the model’s recommendations.

**Figure 9:**
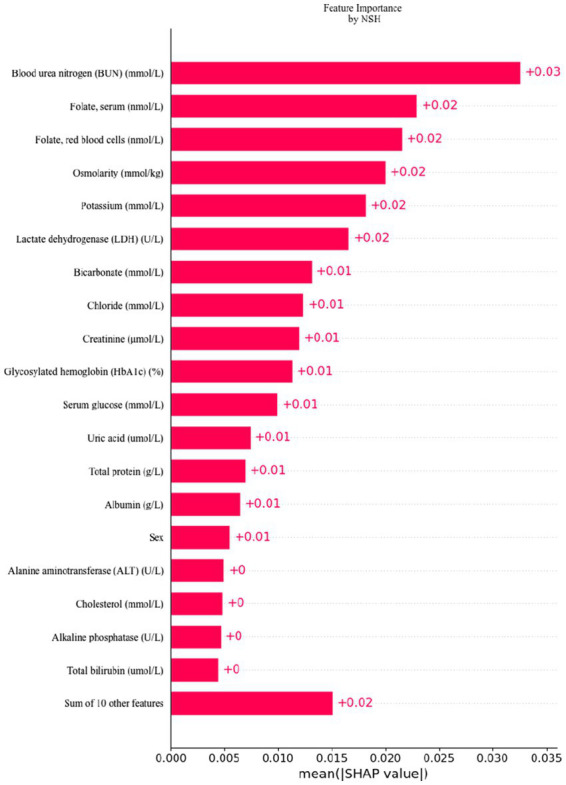
Feature importance ranking by SHAP: this matrix diagram ranks the importance of each covariate in the development of the final predictive model, highlighting which features contribute most significantly to the model’s output.

## 5 Discussion

This study employed LASSO regression alongside multivariate logistic regression to identify key factors associated with cataract risk and to construct a predictive model. Four predictors were evaluated: age, erythrocyte folate (nmol/L), blood glucose (mmol/L), and blood urea nitrogen (mmol/L). Additionally, a logistic regression model was developed using the identified factors. The predictive model demonstrated excellent discriminatory power, calibration, and clinical utility, and was visualized through a nomogram, allowing easy interpretation of the predicted probability.

The LASSO regression technique was used to select independent risk factors for the purpose of modeling and predicting variables of various types. The application of penalized regression reduced the coefficients of less significant independent variables to zero, thereby enhancing model stability. Numerous studies have also employed machine learning techniques to improve and train nomogram-based prediction models for accurately predicting the survival outcomes of patients with breast and colon cancer (24, 25). Multiple factors have been reported to influence cataract development, including socio-demographic and lifestyle factors (4), nutrient intake (12), blood components (26), and genetic predispositions (27). The primary objective of this study was to investigate the influence of blood components and age on cataract formation, visualizing the results through a nomogram. To our knowledge, this is the first study to utilize a nomogram to illustrate cataract risk. Multivariate logistic regression analysis in this study revealed statistically significant differences across four variables: blood urea nitrogen (mmol/L), serum glucose (mmol/L), RBC folate (nmol/L), and age. Each of these factors will be discussed in detail in the subsequent sections. The study by C. Y. Huan et al. identified a significant correlation between chronic kidney disease (CKD) and an increased incidence of both prevalent and incident cataracts (28). B. E. Klein et al. suggested that elevated serum blood urea nitrogen (BUN) and creatinine levels are associated with the development of posterior subcapsular cataracts in continuous models (29). These findings, consistent with those of the present study, suggest that elevated blood urea nitrogen is a risk factor for cataract development, with an odds ratio of 1.042 and a 95% confidence interval of 1.009–1.074. Several potential mechanisms are outlined below. The initial hypothesis suggests that chronic hypocalcemia in patients with chronic kidney disease may disrupt glucose metabolism in the lens (30). The interplay between calcium levels, glucose metabolism, and lens health is complex. Nevertheless, in this study, blood urea nitrogen exerted a more pronounced influence on cataract formation compared to calcium and glucose, likely due to its impact on lens osmolarity, thus promoting cataract development. The second hypothesis proposes that elevated blood urea nitrogen levels disrupt enzymes critical to lens metabolism. Oxidative stress is widely acknowledged as a major contributor to cataract formation, with antioxidant enzymes like glutathione synthase, thioredoxin reductase, glutathione reductase, and thioltransferase playing pivotal roles in slowing cataract progression (31). Elevated blood urea nitrogen may impair the activity of these antioxidant enzymes, thereby accelerating cataract progression. These potential mechanisms require experimental validation. Kang K H, Shin D, Ryu I H, et al. found that fatty liver disease (FLD) may serve as an independent risk factor for cataracts (32), likely due to its role in systemic metabolic disorders. These systemic disorders, often resulting from dyslipidaemia and chronic inflammation linked to FLD, can disrupt metabolic processes throughout the body. One such disruption involves altered biochemical indices, including elevated blood urea nitrogen (BUN) (33). Elevated BUN levels may indicate impaired renal function or increased protein catabolism, both of which could contribute to cataract pathophysiology by promoting oxidative stress and osmotic imbalances in the lens. Therefore, our findings suggest that the heightened risk of cataracts observed in patients with FLD may be mediated, at least in part, by elevated blood urea nitrogen levels. This underscores the need for further investigation into the specific mechanisms connecting FLD, abnormal biochemical markers, and cataract formation, as well as the potential for targeted interventions to mitigate these metabolic disruptions. L. Li et al. identified a significant increase in the likelihood of cataract development among individuals diagnosed with type 2 diabetes mellitus (34). According to this study, elevated glucose levels were associated with an increased likelihood of cataract development. The role of folic acid as a risk factor for cataracts remains debatable. A. Tan et al. showed the 5-year PSC incidence with no significant associations with homocysteine, B12, and folate (35). But C. Ma et al. showed lower serum folate levels in cataract patients compared to controls (36). In addition, W. G. et al. found that in a randomized, double-masked, placebo-controlled trial, combined folic acid, vitamin B6, and vitamin B12 supplementation may increase the risk of cataract extraction surgery (37). The results of W. G. et al. are similar to ours in that folate (nmol/L) was higher in cataract patients compared to non-cataracts, and higher RBC folate (nmol/L) may be a risk factor for cataracts, but to a lesser extent with an OR close to one. Among the previous studies, folic acid supplementation was considered protective against cataracts (38). Tan, A. and colleagues utilized posterior subcapsular cataract (PSC) as the outcome measure in a 5-year follow-up study, revealing that elevated homocysteine levels (per SD; OR 1.17; 95% CI 1.00–1.37) and reduced folic acid levels (per SD; OR 1.24; 95% CI 0.99–1.56) were associated with a higher prevalence of PSC36. Ma, C., Liu, Z., Yao, S., Hei, L., and Guo, W. prospectively recruited 60 patients with senile cataracts and 58 age-matched healthy controls, finding that blood folate levels were significantly lower in cataract patients than in healthy controls. Kuzniarz, M. and Mitchell, P. conducted a cross-sectional study with 2,873 participants, categorizing cataract types and concluding that folic acid supplementation had a protective effect against cortical cataracts. Despite differences in methodology, all three research teams consistently found that cataract patients had lower folic acid levels and that folic acid supplementation may confer a protective effect against cataracts. However, the findings of Christen, W. G. and colleagues were unexpected. In contrast to the previous three studies, Christen, W. G. and colleagues conducted a randomized, double-blind, placebo-controlled trial under more stringent conditions, involving 3,925 participants and yielding more robust results over a follow-up period of up to 7.3 years. In this large-scale randomized trial of women at high risk for cardiovascular disease, daily supplementation with folic acid, vitamin B6, and vitamin B12 had no significant impact on cataract incidence but may have increased the risk of cataract extraction. The findings of Christen, W. G. and colleagues, which aligned with our results that also focused on cataract removal, indicated a facilitating effect of folic acid with an OR close to 1 (95% CI 1.0001–1.0004). The aforementioned studies varied considerably in design, encompassing both observational studies and randomized controlled trials (RCTs). The study populations also differed in demographics, baseline health conditions, and genetic predispositions, all of which may have influenced the observed association between folic acid levels and cataract risk. For example, both this study and the work by C. Ma et al., which used cataract surgery as the outcome measure, reached the same conclusion: higher folic acid levels increased the risk of cataract extraction. These findings underscore the need for longitudinal studies with extended follow-up periods to comprehensively assess the role of folic acid in cataract development. Given the findings of this study, we recommend exercising caution when considering folic acid supplementation as a means to delay the onset of cataracts. It is well established that age is a major determinant of cataract development and requires little further discussion (6).

This study has several limitations. In the absence of direct lens assessments in the NHANES dataset, cataract surgery was used as a surrogate marker for cataract occurrence. A similar approach has been employed in previous epidemiological studies11. However, the distinctions between the two approaches should not be overlooked. The decision to undergo cataract surgery is influenced by a multitude of factors, including cataract severity, visual acuity, ocular measurements, the surgeon’s clinical expertise, and patient preferences (39). The decision to opt for cataract surgery is heavily contingent upon financial resources (40), which also shape health literacy and behavioral patterns, subsequently influencing blood biochemical markers (41). When cataract surgery is employed as an outcome measure, this economic disparity introduces significant selection bias (42). Individuals with higher disposable income and better access to healthcare are more likely to undergo regular ophthalmologic evaluations, facilitating early cataract detection and timely intervention. Conversely, individuals from lower socioeconomic backgrounds frequently delay or forgo surgery due to financial barriers, leading to pronounced disparities in health outcomes. Furthermore, health literacy—the capacity to access, interpret, and comprehend essential health information—tends to be higher in wealthier populations. Wealthier individuals are generally more proactive in managing their health, frequently engaging in preventive behaviors such as regular medical check-ups and strict adherence to medical advice. This often results in more favorable biochemical profiles (e.g., better glycemic control), potentially influencing study outcomes. The direct correlation between socioeconomic status and improved access to nutrition, healthcare, and healthier lifestyles is well-documented (43). Populations of lower socioeconomic status typically present with more abnormal biochemical markers and a higher prevalence of severe cataracts (44). Failure to account for these socioeconomic factors may lead to an overestimation of the impact of biochemical markers on cataract risk. This overestimation may partly arise from the fact that individuals of lower socioeconomic status are more likely to adopt unhealthy lifestyles, such as poor diets and lack of exercise, and face limited access to quality healthcare. Consequently, the observed association between biochemical indicators and cataract risk may be confounded by underlying socioeconomic conditions. Additionally, cataract surgery reflects a relatively advanced stage of the disease, and the relationship between early lens opacity and biochemical markers could not be assessed using NHANES data. Furthermore, the data derived from cataract surgery do not allow for differentiation between distinct types of cataracts in individual patients.

Nevertheless, several limitations exist in this study. The risk factor analysis did not account for potential variables such as patients’ daily living environments and dietary habits, which were not integrated into the predictive model. Incorporating these factors would likely enhance the model’s predictive accuracy and overall performance. This study was conducted retrospectively at a single center, and the predictive validity of the model was not assessed through external validation. This study was a retrospective analysis conducted at a single center. The predictive validity of the model was established using internal validation methods; however, external validation was not performed. It is important to note that while the model shows promise based on its internal validation, the lack of external validation limits our ability to generalize the findings to other settings or populations. Future research will focus on validating the model using large datasets from multiple regions and centers to enhance its predictive accuracy and broader applicability.

## 6 Conclusion

This study identified blood urea nitrogen (mmol/l), serum glucose (mmol/l), erythrocyte folate (mmol/l), and age as significant risk factors for cataracts, and subsequently developed a cataract risk prediction model. This model demonstrated strong predictive accuracy and clinical applicability, offering clinicians a valuable tool for early and accurate diagnosis. Cataract progression may be delayed by lowering blood urea nitrogen, serum glucose, and erythrocyte folate levels through lifestyle modifications and dietary improvements.

## Data Availability

The datasets presented in this study can be found in online repositories. The names of the repository/repositories and accession number(s) can be found here: https://www.cdc.gov/nchs/nhanes/ and in the article/Supplementary material.
